# Subjective perceived impact of Tai Chi training on physical and mental health among community older adults at risk for ischemic stroke: a qualitative study

**DOI:** 10.1186/s12906-017-1694-3

**Published:** 2017-04-20

**Authors:** Guohua Zheng, Zhenyu Xiong, Xin Zheng, Junzhe Li, Tingjin Duan, Dalu Qi, Kun Ling, Lidian Chen

**Affiliations:** 1 College of Health Information Technology and Management, Shanghai University of Medicine & Health Sciences, Shanghai, 201318 China; 2 College of Rehabilitation Medicine, Fujian University of Traditional Chinese Medicine, Fuzhou, 350122 China; 3 Department of Physical Education, Fujian University of Traditional Chinese, Medicine, Fuzhou, 350122 China; 4 Fujian Key Laboratory of Rehabilitation Technology, Fujian University of Traditional Chinese Medicine, Fuzhou, 350122 China

**Keywords:** Tai chi, Qualitative study, Perceived benefit

## Abstract

**Background:**

Evidence from quantitative studies suggest that Tai Chi produces a variety of health-related benefits, but few qualitative studies have investigated how older adults perceive the benefit of Tai Chi. The objective of the current study was to qualitatively evaluate the perceived benefits of Tai Chi practice among community older population.

**Methods:**

This study was conducted with participants from a trial examining the effects of a 12-week Tai Chi training on ischemic stroke risk in community older adults (*n* = 170). A total of 20 participants were randomly selected from a convenience sample of participants who had completed 12-week Tai Chi training (*n* = 68) were interviewed regarding their perceived benefit on physical and mental health and whether Tai Chi exercise was suitable for the elderly.

**Results:**

All participants agreed that Tai Chi training could relax their body and make them comfortable. Most of them thought Tai Chi training could promote physical health, including relieving pain, enhancing digestion, strengthening immunity, enhancing energy and improving sleep quality, enhancing their mental and emotional state (e.g. improving mood and reducing anxiety, improving concentration and promoting interpersonal relationship). Most of participants also agreed that Tai Chi exercise was appropriate for community older people. Three primary themes emerged from content analysis: Improving physical health; Enhancing mental and emotional state; Conforming with the request of the elderly.

**Conclusion:**

The findings indicate that regular Tai Chi exercise may have positive benefits in terms of improved physical health and mental state among community elderly population, and may be useful and feasible body-mind exercise to community elderly population for its positive effects and advantages.

**Trial registration:**

ChiCTR ChiCTR-TRC-13003601. Registered 23 July 2014.

**Electronic supplementary material:**

The online version of this article (doi:10.1186/s12906-017-1694-3) contains supplementary material, which is available to authorized users.

## Background

Tai Chi is a mind-body exercise that originated in China as a martial art, and has been practiced widely for centuries, primarily in the elderly population. Tai Chi is also known as Taiji chuan, which translates to “supreme ultimate fist;” its conceptderives derived from the combination of both Taoist and Confucian Chinese philosophy, where it represents the fusion of yin and yang into a single ultimate (i.e. Taijin). As a comprehensive exercise, Tai Chi combines meditation with a series of slow and graceful continuous movements, breathing, and relaxation [1, 2]. Evidence from quantitative studies suggest that Tai Chi produces a variety of health-related benefits for older individuals by improving balance, promoting cardiorespiratory functions, enhancing psychosocial well-being, improving muscle strength, reducing the risk of falling, and enhancing quality of sleep [3–8]. Regular Tai Chi practice may also be effective for managing multiple chronic conditions, including pain, depression, stress, anxiety, hypertension, dyslipidemia, arthritis, cardiovascular disease, and type 2 diabetes [9–12]. As a complex, multi-component exercise that integrates numerous physical, cognitive, and ritualistic components, the practice of Tai Chi should be practiced in harmony with the *Taiji* philosophy by utilizing and manipulating *qi* via *Taiji.*

Quantitative research studies produce outcomes that can be readily captured by conventional instruments; however, these methods may be unsuitable to analyze Tai Chi. Recently, there has been increasing recognition of the role qualitative approaches can play in the development and evaluation of complex interventions [13, 14]. Although some qualitative studies have evaluated the feasibility, adherence, and experience of Tai Chi among patients with rheumatoid arthritis or osteopenia [15, 16], to date, few qualitative studies have investigated how older adults perceive the benefit of Tai Chi, particularly in those with no previous Tai Chi experience. Here, we conducted a qualitative study to explore the individual perceived benefits of the Tai Chi practice in older adults.

## Methods

### Study design

The current qualitative study was conducted in a randomized controlled trial examining the protective effects of a 12-week Tai Chi training on ischemic stroke risk in a community older adults at risk of ischemic stroke (ChiCTR-TRC-13003601) [17]. The data were collected using semi-structured interviews (see description in the Additional file 1). Interviewees freely reported their own views and understanding regarding their perception of the Tai Chi intervention.

### Samples

Participants were recruited through community advertisements, posters, and leaflets at a free clinic in three community centers in the Gulou district of Fuzhou city in China. The inclusion criteria were as follows: male or female between 55 and 75 years old at risk of ischemic stroke (Individual at high risk of ischemic stoke should meet two items from items 1–7 or item 8: ①Current high blood pressure (systolic/diastolic pressure ≥ 140/90 mmHg) or taking antihypertensive drugs; ②Atrial fibrillation; ③Currently smoking (at least one cigarette each day for 1 year); ④Dyslipidaemia; ⑤Diabetes mellitus; ⑥Obvious overweight or obesity (body mass index (BMI) ≥24 kg/m2); ⑦A family history of stroke (a stroke history in 3 generations); ⑧A history of transient ischemic attack.) with no language barriers, high articulation, and no regular exercise within the past year (regular exercise defined as any exercise or physical activity lasting more than 3 months with a frequency of 30 min or more one to three times or more per week.)

A total of 170 participants were enrolled and randomly allocated to either the Tai Chi training group or the control group at a 1:1 ratio. In the Tai Chi training group, 68 participants completed 12-week Tai Chi training, while 17 other participants dropped out. Using the convenience sample of 68 participants who completed the 12-week Tai Chi intervention, we then randomly selected 20 participants to participate in the semi-structured interviews.

### Intervention

The 24 forms simplified Tai Chi Chuan based on Yang style, which has been recommended as a popular, healthy sport by the Chinese National Sports Commission since 1956, was applied to the Tai Chi training group. Participants were gathered at the community center and practiced Tai Chi Chuan with five days per week and an hour one day for 12 weeks. Two certified instructors conducted Tai Chi training and the ‘meditation through movement’ art of Tai Chi Chuan, including relaxation, breathing skills, and coordinated movement of the arms, legs, and body, as well as the philosophic aspects of Tai Chi.

### Date collection

The semi-structured interviews were conducted in July 2014 by the second author (LJZ) who is trained in qualitative studies. The interviews aimed to explore the perceived effect of Tai Chi on the physical and mental state, including the subjective experience of training, perceived changes in body and mind, and the suitability of Tai Chi for the elderly. A prepared interview guide was designed to draw out the experience and was used in face-to-face interviews to cover three main topics: the experience of practicing Tai Chi, changes on the physical and emotion state, and suitably for the elderly; the first question was, “Can you tell me how Tai Chi training affected you?”

Interviews were performed flexibly to ensure sensitivity. Each interviewee was encouraged to express their viewpoints and experiences freely and truly. The second interview was conducted to avoid loss of any additional information, as iterative questioning is crucial to build credibility and trustworthiness. The approximately 40-min interviews were conducted at the participants’ homes under quiet conditions. All interviews were recorded by digital recording pen and transcribed verbatim by the third author (ZX).

### Data analysis

Interview transcripts were analyzed in accordance with Granheim and Lundman’s qualitative content analysis [18]. First, in order to obtain an overall impression, the transcribed materials were read several times thoroughly. Then, sentences or phrases were condensed and divided into the meaning units. The meaning units were further condensed, abstracted, and conceived into the codes. All codes were put together and compared, based on differences and similarities, and the codes with similar content were sorted into sub-categories and then into categories. Finally, the themes were formulated. To enhance authenticity, all authors were involved in the discussion and were consulted until a consensus was reached during the analysis procedure, and the meaning units, codes, categories, and themes were inspected to ensure validity (Table 1).

**Table 1:** Participants’ characteristics

Characteristics	Intervention group *n* = 20
Female	14
Male	6
Age (range year)	62(50–70)
Occupation	
Pensioner	19
Working	1
Education	
≥ 12 years	15
<12 years	5
Risk factors of ischemic stroke	
Hypertension	12
Dyslipidemia	13
Diabetes mellitus	2
Overweight or obesity	12
Family histroy of stroke	5
Medication	
Yes^a^	12
No	8

Values are median (range) or number

^a^Including metoprolol, atorvastatin, amlodipine, acarbose and so on

### Ethical considerations

This study complied with the Helsinki Declaration and was approved by the Medical Ethics Committee of The Affiliated People’s Hospital of Fujian University of Traditional Chinese Medicine (No.2013–020-02). All participants provided written informed consent and were assured confidentiality and anonymity.

## Results

### Sample characteristics

Demographic characteristics of participants are shown in Table 2. Twenty participants (14 women and 6 men) ranged from 50 to 70 years with a median age of 62 years. Most participants suffered from hypertension (12, 60%), dyslipidemia (13, 65%), and obesity (12, 60%). 2 participants (10%) were diagnosed with diabetes mellitus, and 5 participants had a family history of stroke. Among these participants, most (19, 95%) were retired, 15 (75%) had received more than 12 years of education, and 12 (60%) took regular medications for chronic conditions.

**Table 2:** An analysis example of meaning units, Condensed meaning units, Codes and sub-category according to Graneheim and Lundman [18]

Meaning units	Condensed meaning units	Codes	Sub-category
‘My appetite had slowly become better since I practiced Tai Chi…In the past years, I always fell lack appetite’.	My appetite became bettre than before.	Improving appetite	Enhancing digestion
‘It seemed hemorrhoids would be better. ... Previously, I felt painful for defecation when the weather was dry, ... My anus was uncomfortable. It was better after practicing Tai Chi this year’.	I felt easier for defecation.	Promoting defecation	
‘I used to have flatulence after meal. …..However, it’s much better now’.	Less flatulence after meal	Relieving flatulence	

### Primary themes

Data analysis showed that participants who practiced Tai Chi regularly experienced greater benefits. Three vital themes related to their experiences and subjective perceived benefit over a period of practicing Tai Chi were described as following: (1) improved physical health, (2) enhanced mental and emotional state, (3) conformation with the request of the elderly. Themes and subthemes are shown in Table 3.

**Table 3:** Overviews of subthemes and themes

Subthemes	Themes
Feeling comfortable and relaxed and Relieving pain	Improve physical health
Enhancing digestion	
Strengthening immunity	
Enhancing energy	
Alleviating breathless	
Improving sleep quality	
Improving mood and reducing anxiety	Enhancing mental and emotional state
Improving concentration	
Promoting interpersonal relationship	
Easily arousing interest	Conforming with the request of the elderly people
Appropriate amount of exercise	
Quick warm-up	
Less limitation on exercise place	

### Theme 1: Improved physical health

All participants experienced changes in their physical condition. Tai Chi training improved their physical health across various aspects.

### Feeling comfortable and relaxed and relieving pain

All participants described Tai Chi as an excellent exercise that helped exercise their whole body, including the head, neck, waist, hands, and feet. They also felt more comfortable and relaxed after practicing Tai Chi each day. Five participants said that Tai Chi helped relieve their neck soreness, muscular soreness, and lower back pain.

“I had a neck soreness for a long time. I felt better after practicing Tai Chi. Since I helped my son take care of his child, I didn’t have time to practice for a while. My neck soreness started bothering me again. Then, I started practicing Tai Chi again, my neck soreness was alleviated.” (ID15, female, 58 year old, hypertension and hyperglycemia).

### Enhancing energy

Fourteen participants reported that their strength improved through practicing Tai Chi. In the past, they became tired and felt exhausted due to their poor physical condition; however, they experienced an obvious improvement in strength after practicing Tai Chi.

“In the past I was not wide awake and dozed in the morning sometimes; I was easily fatigued when I did housework. I have more energy and feel tireless now.” (ID 5, female, 58 years old, hyperlipidemia and obesity).

### Improving sleep quality

Seven participants reported that they fell asleep easily and enjoyed abundant sleep after Tai Chi. Before training, they had sleep complaints, such as difficulties falling asleep, insomnia, short sleep duration, and being easily awoken.

“I fell asleep soon when I laid down. It was hard to wake up at midnight. Now, I get a good night sleep. Tai Chi aided restful sleep assuredly. I slept poorly before that. I took an hour to fall asleep, awakened easily in the middle of night, I couldn’t get sleep, and was still in a daze at dawn, then got up.” (ID 8, female, 56 years old, hypertension, hyperlipidemia, and obesity).

### Strengthening immunity

Five participants reported that they seldom felt ill after practicing Tai Chi; They experienced far fewer colds, for example.

“It seemed like there was nothing wrong (with me)...(I) very rarely got colds. I had a fever, and yet I got well next day.” (ID 12, female, 61 years old, hyperlipidemia and obesity).

### Enhancing digestion

Four participants suffered from gastrointestinal problems, such as poor appetite, flatus, and distension. Tai Chi training helped their gastrointestinal problems, including poor appetite, gastric distention, hemorrhoids, and change in stool form (dry or looser).

“My appetite had slowly become better when I started practicing Tai Chi…In the past years, I have had a lack of appetite.” (ID 10, male, 67 years old).

“It seemed that my hemorrhoids would be better... Previously, I felt pain during defecation when the weather was dry...My anus was uncomfortable. It was better after practicing Tai Chi this year.” (ID 16, male, 70 years old, hypertension and a family history of stroke).

“I used to have flatulence after meals...However, it’s much better now.” (ID 11, female, 69 years old, hypertension and dyslipidemia).

### Alleviating breathlessness

One participant reported that Tai Chi training helped alleviate his breathlessness. “There was a slope next to our residential area that we would walk through...In the past, I felt a little breathless going up the slope. Now, I am basically not breathless after I finished Tai Chi training. I thought it still had apparent effect.” (ID 13, female, 65 years old, hypertension and hyperlipidemia)*.*

### Theme 2: Enhancing mental and emotional state

Tai Chi is a mind-body exercise. As a result, apart from physiological effects, it also improves the mental and emotional state. The participants experienced the slow but continuous movements and soft music to obtain “body relaxation and mind calm.” Most participants reported that Tai Chi made them feel calm, pleasurable, and anxiety-free by improving concentration and interpersonal relationships.

### Promoting interpersonal relationship

Most participants (seventeen individuals) said that they prefer practicing Tai Chi together with others instead of practicing it alone so they can share their physical experiences of practicing Tai Chi in a relaxed and pleasant atmosphere.

“It was great that our several sisters practiced together everyday and chatted with each other.” (ID 7, female, 62 years old, hyperlipidemia and obesity).

“It was an enjoyable practicing together with our elderly.”(ID 20, male, 69 years old, hyperlipidemia and hypertension).

### Improving mood and reducing anxiety

Eleven participants reported feeling totally absorbed by training. They felt Tai Chi entered their inner world when they were practicing, and made them relax, calm down, and forget their troubles.

“I quarreled with my family just at very small things sometimes. Now, the feeling is less.” (ID 2, female, 58 years old, hyperlipidemia and obesity).

“I am in a good mood after Tai Chi training every day...” (ID 9, female, 64 years old, hypertension, takes benazepril).

“My daughter used to complain about my bad temper. I was often angry at her. Now, I am more clam and patient.” (ID 8, male, 61 years old, dyslipidemia and smoking).

### Improving concentration

Six participants reported that they were easily distracted when they were doing housework or other work due to poor memory and inattention.

“I was always easily distracted when I was doing something before…Now I feel I can centralize my attention to complete work.” (ID 16, male, 70 years old, hypertension and a family history of stoke).

### Theme 3: More suitable to the elderly

Several types of exercises, including jogging, swimming, hiking, and climbing, are not suitable for older adults. Most participants thought Tai Chi should be recommended as a suitable exercise for elderly people due to the moderate exercise intensity and less limitation on exercise location.

### Appropriate amount of exercise

Compared to other exercises, sixteen participants thought the intensity of Tai Chi was appropriate and acceptable for the elderly because it contains slow and gentle movements rather than intensive movements which make them feel exhausted.

“The slow-movement and moderate exercise of Tai Chi is suitable for the elderly. It’s not like dancing with larger movements that made me tired’. (ID 8, female, 56 years old, hypertension, hyperlipidemia, and obesity).

### Less limitation on exercise location

Twelve participants noted that there was less restriction on where to practice Tai Chi compared to other sports, such that it was not dependent on the weather.

“If I practiced by myself, there was no need to find a large space; I could practice just at home.” (ID 13, female, 65 years old, hyperlipidemia and hypertension).

“If it is raining, we can practice Tai Chi at home…” (ID 16, male, 63 years old, hypertension).

### Easily arousing interest

Ten participants reported Tai Chi as the most interesting exercise because Tai Chi consists of a series of slow, continuous movements, along with breathing and meditation.

“Tai Chi is good exercise. I had no spare time to learn it because of my busy work. Now, I have retired and have nothing to do...I like the graceful movements of Tai Chi’. (ID 6, female, 63 years old, hypertension and obesity).

### Quick warm-up

Eight participants noted how fast they warmed up and sweat after doing Tai Chi for two or three times.

“At the same temperature of 15 or 16 degrees, I practiced Tai Chi twice while my body was warming up. With brisk walking, I need half an hour at least to warm up.” (ID 4, male, 64 years old, hyperlipidemia and hypertension).

## Discussion

Three themes which emerged from the data analysis reflected the experiences of participants as well as the subjective perceived benefits and advantages of Tai Chi. The first theme is that Tai Chi can improve physical health, including alleviating pain and difficulties with respiration, improving immunity and quality of sleep, and enhancing digestion and energy. Tai Chi can also help with relaxation. These findings were similar to those reported in other quantitative studies [19–25]. In one literature review, Peng PW reviewed the role of Tai Chi in a few selected chronic pain conditions including osteoarthritis, fibromyalgia, rheumatoid arthritis, low back pain, and headache. Author reported that Tai Chi seems to be an effective intervention in osteoarthritis, low back pain and fibromyalgia [19]. Another author also reported that Tai Chi is a potentially effective treatment for neck pain in the systematic review [20]. In our study, participants reported that Tai Chi enhanced their quality of sleep, which has also been described by other studies on the effect of Tai Chi on sleep quality in the elderly [21]. One previous study found, as a preventative and rehabilitative practice, an intervention of Tai Chi for 15 weeks produced an increase in varicella-zoster virus specific cell-mediated immunity [22]. This result indicated that Tai Chi is helpful to strengthen immune function, and it is similar to our findings. With regular practice, participants in present study reported feeling energetic and less tired than before the intervention, which was associated with an improvement in strength. Zhou and Song reported that regular Tai Chi training with over 6 months can enhance the muscle strength in community-dwelling population in their studies [23, 24]. The effect of Tai Chi on improvement in aerobic capacity was reported in a previous meta-analysis, [25] which is consistent with our findings as well.

The second theme is that Tai Chi improved mental and emotional states. Previous studies have found that Tai Chi improves mental well-being, including reducing stress, anxiety, and depression [26–28]. Improved concentration appears to be related to the combination of breathing and movements, allowing for “body relaxation and mind calm” [29]. During Tai Chi, participants reported concentrating on postures and movements which helped them forget troubles and unhappiness for that moment. They also reported that Tai Chi helped them enjoy peace and ease which is not typical of their usual exercise.

The third theme is ‘conforming with the request of the elders,’ which explains why Tai Chi is a beneficial exercise for the elderly population. Tai Chi consists of a series of slow, gentle, continuous movements which is appropriate for the physical condition of the elderly; it is also easy to learn and grasp. Tai Chi has been demonstrated to be of moderate exercise intensity, requiring less than 55% of maximal oxygen intake [30], which is similar to brisk walking at a speed of 6 km/h [31]. This exercise intensity is suitable for the elderly population. Another advantage of Tai Chi for elderly adults is the quick warm-up sequence with two or three cycles of movements, which helps provide great benefits to the participants. Tai Chi training can be performed at any quiet, small place, such as in the own house, and the practice is not dependent on the weather.

Some limitations of the study should be discussed. Although qualitative methods are suitable to explore participants’ experiences and their perceived benefits of Tai Chi, the exercise regimen lasted for 12 weeks, which might lead to recall bias in remembering the experience of the moment clearly. Another possible limitation of this study is that all participants were from the same community, so there was a lack of diversity in the sample population. All participants were also at risk of ischemic stroke, with at least one risk factor of ischemic stroke. Therefore, the sample homogeneity may limit generalizability of the findings. Third, no questions regarding any negative effects or challenges of Tai Chi were designed to query participants, and all interviewed participants were those who completed whole 12-week Tai Chi training, so potentially important information about less desirable effects of Tai Chi may be missed. In addition, because Tai Chi is an accepted part of the traditional Chinese culture, it might also be difficult to generalize those findings to the West. The number of interviewees was very small compared to the total number of participants, as the interviewees were enrolled from a purposeful sample (only from the Tai Chi training group) rather than a random sample. These limitations may have led to biased results. Nevertheless, all participants were encouraged to talk in depth about their perspectives on the research topic. Furthermore, we visited the same participant for a second time if additional information was needed. Current qualitative investigations are based on a randomized controlled trial with a rigorous design. Those findings should be helpful to enhance the evidence of effectiveness produced by the RCT or facilitate the feasibility of the RCT itself [32].

## Conclusions

The results of present study suggest that the practice of Tai Chi provides several benefits that improve physical health and mental state among community elderly population at risk of ischemic stroke. Participants reflected a number of benefits, including relieved pain, improved sleep quality, enhanced digestion, strengthened immunity, enhanced energy, relieved breathless, reduced anxiety, improved concentration and promoted interpersonal relationship. In addition, they also expressed the view that Tai Chi was an appropriate exercise for the elderly. This findings indicate that Tai Chi is a beneficial and feasible exercise that improve body-mind health among community elderly population.

## Additional file

Additional file 1: The outlines of interview. (DOCX 14 kb)
