# Supplementary material for: Subjective perceived impact of Tai Chi training on physical and mental health among community older adults at risk for ischemic stroke: a qualitative study
Source: BMC Complement Altern Med. 2017 Apr 20;17:221. doi: 10.1186/s12906-017-1694-3 (PMC5397805; doi:10.1186/s12906-017-1694-3)
Supplement: Additional file 1: — The outlines of interview. (DOCX 14 kb) [file 12906_2017_1694_MOESM1_ESM.docx]

**Appendix**

**The outlines of interview**

Date of interview: _________ ______

Interviewer: ___________ _______ __

Participants: __________ _________

**Introduction:**

- Thanks for coming in for this interview
- Purpose: To explore how you think about Tai Chi training. Hear your experiences of this training program. Learn more about your subjective perceived effect of Tai Chi training on physical and mental health that we may have missed on the physical measurements.
- Confidentiality: Your answers will never be linked to your name. You will remain anonymous. Only study staff will have access to the recordings and transcripts.
- These interviews usually take about 40 mintues.
- If there are any questions that you do not want to answer. Please let me know.
- Do you mind if we tape the interview? Yes/No (turn on recoder)

**1. The narrative about practicing Tai Chi training:**

- Could you tell me how Tai Chi exercise program were for you? (if no spontaneous narrative: Please tell me any stories from the exercise program.)
- Can you tell me how you feel after the Tai Chi exercise?
- What do you think about Tai Chi exercise?

**2. Have you see any changes in your body or mind because of the Tai Chi exercise?**

- Have you felt any changes in your physical or mental aspect?
- Can you narrate these changes in detail?
- Do you think these changes result from Tai Chi exercise?
- How do you think Tai Chi exercise work?

**3. Is Tai Chi exercise suitable for elderly?**

- Do you feel the difficulty to learn the postures of Tai Chi exercise?
- Can you understand the relationship the postures of Tai Chi exercise and your breath?
- Do you feel tired after completed the Tai Chi exercise each day?
- Can you continue to practice Tai Chi exercise in your future life?

**4. Closure:**

- Do you have any questions about Tai Chi exercise program?
- How was this interview for you?

**5. Observational notes (interviewer: Please record any body language, other cues):**
